# Insights Into Sibling Relationships and Longevity From Genetics of Healthy Ageing Nonagenarians: The Importance of Optimisation, Resilience and Social Networks

**DOI:** 10.3389/fpsyg.2022.722286

**Published:** 2022-05-06

**Authors:** Jennifer Nicola M. Rea, Katarzyna Milana Broczek, Elisa Cevenini, Laura Celani, Susanne Alexandra J. Rea, Ewa Sikora, Claudio Franceschi, Vita Fortunati, Irene Maeve Rea

**Affiliations:** ^1^Department of Primary Care and Population Health, University College London, London, United Kingdom; ^2^The Polish Society of Gerontology, Warsaw, Poland; ^3^CIG-Interdepartmental Centre ‘L. Galvani’, University of Bologna, Bologna, Italy; ^4^Belfast School of Art, Ulster University, Belfast, United Kingdom; ^5^Nencki Institute of Experimental Biology, Polish Academy of Sciences, Warsaw, Poland; ^6^School of Medicine, Dentistry and Biomedical Sciences, Queen’s University Belfast, Belfast, United Kingdom; ^7^School of Biomedical Science, Ulster University, Coleraine, United Kingdom; ^8^Belfast Health and Social Care Trust, Belfast, United Kingdom

**Keywords:** nonagenarian siblings, nonagenarian sibling relationships, Gold typologies, coping strategies, optimism, resilience, social activities engagement

## Abstract

Understanding how to “Age Longer and Age Well” is a priority for people personally, for populations and for government policy. Approximately ten percent of nonagenarians reach 90 years and beyond in good condition and seem to have a combination of both age-span and health-span. However, the factors which contribute to human longevity remain challenging. Culture is a shared system of learning ideas, feelings, and survival strategies. It has a strong influence on each person’s psychological development, behavior, values and beliefs. Nonagenarians have rich life experiences that can teach us much about aging well; they are rich reservoirs of genetic, lifestyle and psychological information which can help understanding about how to live longer and better. Sibling or trio nonagenarians are important sources of family beliefs and behaviors upon which individual personalities may have been built. Their personal family histories and narratives are powerful tools that help to determine familial traits, beliefs and social behaviors which may help establish factors important in the siblings’ longevity. Using purposefully selected subjects, recruited to the Genetics of Healthy Ageing (GeHA) project in four European countries, this research used the simple life story and qualitative research methods to analyze contrasting and distinctive questions about the interface between the psychological and social worlds as presented in the nonagenarian siblings’ insights about their longevity. Their stories aimed to give better understanding about which psychological aspects of their common life journey and the degree of emotional support in their sibling relationships may have supported their paths to longevity. The most universal finding in each of the four European countries was that nonagenarians demonstrated high positivity, resilience and coping skills and were supported in social networks. Around this theme, nonagenarians reported “being happy,” “always cheerful,” “never melancholy” and having a contentment with a “rich life” and family relationships which fits with accumulating evidence that life satisfaction comes from a perceived self-efficacy and optimism. Most sibling relationships in this study, when analyzed according to the Gold classification, fit the “congenial” or “loyal” relationship type – demonstrating a healthy respect for the others’ opinion without overt dependence, which may help individual coping and survival mechanisms.

## Introduction

Today many people can expect to live into their 80s, 90s, and even beyond 100 and new advances in medical and biomedical technology and our increasing understanding about how to slow aging, make it likely that by 2050, most women will live to be at least 90 years of age and men 85 years (The Millbank Quarterly, 2010); that is a decade longer than most current government predictions and has important and far-reaching implications for all aspects of society. However, few research studies have involved nonagenarian siblings and their personality, psychological profiles and attitudes to longevity. In our research we have used a life story approach with nonagenarian siblings to assess how their siblinghood and psychological profiles may have contributed to the quality of their longevity.

Psychological wellbeing in aging and longevity is of increasing interest alongside genes, which have also been a focus in longevity science (Perls et al., 2000; Kojima et al., 2004; Deelen et al., 2011, 2014; Beekman et al., 2013; Rea et al., 2015; Revelas et al., 2018). Family clusters of nonagenarian and centenarian siblings who show both exceptional age-span and health-span are likely to have inherited not only facilitatory gene groups, but also have nine decades of life experiences and behaviors which have interacted and imprinted their personalities and psychological profiles. There is evidence, that subjective wellbeing and health are closely linked to both age and mortality. In positive psychology research, understood as well-being, happiness and mental health, three aspects of subjective wellbeing are recognized -*evaluative wellbeing* or life satisfaction, *wellbeing which focuses on emotional feelings* of happiness, sadness, anger, stress, and pain (Kahneman et al., 2003), and *wellbeing related to personal self-realization*, with a focus on personal resources, resilience, life meaning and purposefullness (Waterman et al., 2010; Ryff, 2014).

A meta-analysis involving 26 studies, showed that positive affect (e.g., emotional well-being, positive mood, joy, happiness, vigor, energy) and positive dispositions (e.g., life satisfaction, hopefulness, optimism, sense of humor) were associated with reduced mortality in healthy population studies, and with reduced death rates in patients with diseases such as renal failure and human immunodeficiency virus-infection (Chida and Steptoe, 2008). Similarly, data collected as an adjunct to the international HAPPIE project (2006–2008), in Lithuania, demonstrated that psychological well-being was a predictor of longevity, even after correcting for well-recognized risk factors such as age, education, cardiovascular diseases, social status, marital status, lifestyle and biological factors (Tamosiunas et al., 2019). In a follow-up series of analyses of wellbeing across age-groups in the English Longitudinal Study of Aging, Steptoe et al. (2015), identified that wellbeing and optimism were associated with increased survival, reporting that 29.3% of people in the lowest wellbeing quartile died during an 8.5-year follow-up, compared to 9.3% in the highest quartile of wellbeing. In further life evaluation assessment studies (broadly defined as “happiness” with life or “life satisfaction”), involving European, American, Asian, and Latin American cross-sectional surveys over several time periods, there was replication of prior findings of a U-shaped association between age and wellbeing with the nadir at middle age and higher wellbeing in younger and older adults, in both developing and advanced countries (Blanchflower and Oswald, 2008; Blanchflower, 2021). In later studies the U-shape curve was less clearly identified in analyses of longitudinal data from Britain, Germany and Australia, after correcting for other effects and findings remain under discussion (Frijters and Beatton, 2012; Lachman, 2015; Galambos et al., 2020).

Relatively little research concerns older siblings, either as a sibling pair, or each as a sib in their own right. Sibling relationships are often amongst an individual’s longest-lasting relationships throughout life. Most sibling research studies have focused on children and adolescents with only a few in older adults (Ross and Milgram, 1982; Cicirelli, 1995, 1996; Van Volkom, 2006; Conger and Little, 2010), and little, if any, in siblings aged 90 or older. In adolescent life shared by siblings, sibling relationships have been demonstrated to have significant psychological influences in each other lives (Volling, 2003; Whiteman et al., 2011; McHale et al., 2012), with research increasingly interested in understanding how sibling hierarchy and relationships can affect behaviors in adulthood. The relationship between nonagenarian siblings has not been previously described or self-assessed. It was therefore of interest to use the five typologies- “Intimate,” “congenial,” “loyal,” “apathetic,” and “hostile” from the Gold (1989) classification to characterize the nonagenarian intra-sibling relationships.

In this research the aims were to

(1) assess if the five typologies described by the Gold (1989) classification in younger sibling pairs were also identifiable in GeHA nonagenarian sibling pairs.
(2) gain knowledge about GeHA nonagenarian siblings’ self-insights into their longevity in terms of their psychological personalities, behaviors and social networks, through a simple life-story method using qualitative research methods.

## Subjects and Methods

The subject group was 20 sibling pairs plus 2 trio sibling (46 nonagenarians) participants of the Genetics of Healthy Ageing (GeHA) EU Project (Franceschi et al., 2007), the linked EU ACUME2 Socrates project (Rea et al., 2015) and Super Vivere study (Rea and Rea, 2011). These 90-year-siblings who lived in their own homes in Italy (five pairs), Finland (four pairs and one trio), Northern Ireland (six pairs and one trio), and Poland (five pairs), had shown an interest, willingness and enthusiasm to be involved in the life-story aspects of the ACUME2, Super Vivere and Beyond 90 Together projects, Table 1. Using purposefully interested subjects such as those already recruited to the GeHA project is in keeping with the theory of qualitative sampling (Patton, 1990; Benoot et al., 2016; Crossman and Nicki, 2020), which suggests that such a sample is likely to include rich cases, selected based on characteristics of a population and the objective of the study, and from which important research information would be likely to be gleaned.

**TABLE 1:** Comparative age, sex and male/female, female/female, male/male dyads and trios for nonagenarian siblings in Italy, Poland, Finland, and Northern Ireland.

	Italy	Poland	Finland	N Ireland
Age (years)*	95(96)	93 (93)	94 (93)	95 (95)
Number	10	10	11	15
Female/Male	9F/1M	9F/1M	9F/2M	9F/6M
Pairs	5	5	4	6
Trios	0	0	1	1
Dyads (F/F)	4	4	3	4
Dyads (M/F)	1	1	2	1
Dyads (M/M)	0	0	0	1
Trios	0	0	FFF	MFF
Single	1	1	2	4
Married	9(7c)	9(7c)	9(6c)	11(10c)

*, mean age; (), median; M, male; F, female; s, single/unmarried; c, number of children; (c), number married with children.

### Telling Their Stories

The Simple Life Story method was the means whereby sibling pairs told their stories. The methodology was simple, brief, and had been used previously to allow elderly people to tell their life story effectively (Ganzevoort and Bouwer, 2007). The interviews were held separately, except where participants lived together and wished to be interviewed together. Because the research team was already known to each of the participants and their families, an easy rapport was readily established.

### The Question “What Do You Think Makes You a Survivor”?

Beyond the simple life story, the participants were asked – What do you think makes you a survivor? Some participants during this phase chose to expand parts of their life story and this added depth and detail which were seen as important to the participants, and these were included in the narrative pieces.

### Photographs

With participant written consent, each sibling participant or pair of sibling participants was photographed in the home environment by the research photographer with his/her agreement for use in educational material or in exhibitions, focused on improving public and personal understanding about healthy aging and longevity (SAJR).

### Ethical Permissions

Written consent for audio recording of the life stories and photographs was given by each involved participant. Ethical Consent was obtained through the Research Ethics Committee Northern Ireland (ORECNI), 08/NIR03/42 and by Queens University Belfast. The consent forms were translated into Italian, Finnish, Polish as well a English, with information about the project given to participants in their own language, before the research project commenced.

### Narrative and Typological Analysis

The word narrative comes from the Latin meaning “knowing” and is often considered to be the same as a story (Randall, 2001; Riessman, 2008). Storytelling or the “knowing” or “showing” of our selves is fundamental to all peoples and cultures. Storytelling seems therefore to help collect and assess personal self-knowledge and improved understanding of how ideas and beliefs are transmitted down through families, generation and communities (Ambrosini and Bowman, 2001; Linde, 2001).

### Narrative Analysis

We used the multi-dimensional analysis of narrative suggested by Barclay (1996) which builds on four levels in narrative: the first level concerns the process of narrating; the second listens to the linguistic characteristics of the narrative; the third determines themes and storylines in the topics and the fourth is concerned with the overarching plot.

### Transcription to Text and Preliminary Analysis

The audio material was first transcribed into written text by third party researchers with bilateral language skills. An initial analysis by 2 independent researchers working separately (IR and JR) gave the “essence” of the timeline, the context, and the happenings within each life story (Lieblich et al., 1998). At a second reading, the sense of any “coherence” between the sibling life stories and answers was assessed. Beyond this, some sense of the overall themes began to emerge within each person’s story, between the nonagenarian sibling pairs or trios, and within the nonagenarian group as a whole were identified, reviewed and reconsidered by two researchers working independently, toward saturation of data (IR and JR).

### Listening to Audio Files

The audio files of each life story was listened to, with attention to the quality of the voice, the intonation, the timbre, and the sense of spiritedness, optimism or sadness. This gave a whole different sense of personhood to the textual interview and gave a further dimension which helped to shape an understanding of the emotional contexts of relationships, war memories, loss and happpiness (Frank, 1998).

### Identifying Themes and Typologies

The answers to the question about survivorship were collected together and grouped into major thematic categories (Luborsky, 1994). Additional information, such as family details about uncles or cousins who lived long, that emerged in the life-story was also identified. Main themes were identified using a “grounded theory” technique from which hypotheses emerge, and which are formulated from the emerging data (Strauss and Corbin, 1990).

### Analysis of Nonagenarian Sibling Relationships Using Gold Typologies

Sibling life-stories and questions were used to assess the relationships between 13 pairs of siblings and 2 groups of trio siblings, using the five typologies described by the Gold (1989) classification. In this classification five sibling types of relationship were described which were characterized as “Intimate,” “congenial,” “loyal,” “apathetic,” and “hostile.” In order to help assess the sibling relationships more fully, the researcher asked two additional questions including

‘**Do you and your sibling share coping mechanisms’**? and ‘**How alike are you to your sibling**’ (brother or sister)? Some participants during this phase chose to expand parts of their life story and this added depth and detail to help characterize the sibling relationship more fully.

## Results

### Nonagenarians Narrative Insights About Their Longevity

In response to the question ‘**What Do You Think Makes You a Survivor**’? three main themes were identified by GeHA nonagenarians as important factors in their longevity.

(1) Positive Attitudes
(2) Resilience
(3) Social networks

Each will be discussed briefly in the context of some of the present-day evidence linking the themes to scientific evidence and relating these life-style behaviors to health benefits.

#### Positive Attitudes

**FIGURE 1:**
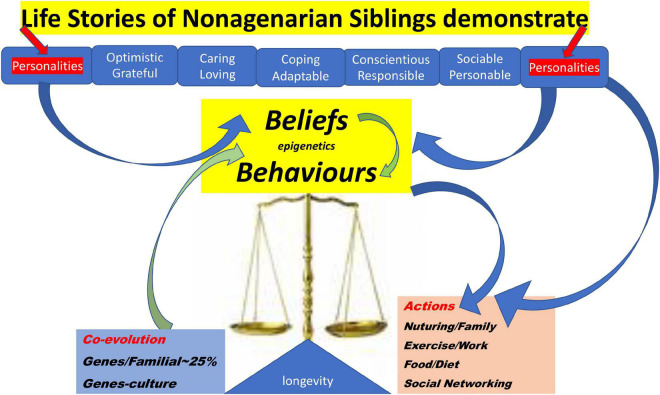
Diagram demonstrating Nonagenarian Sibling Themed Insights into their Longevity, and pathways linking Genes, Beliefs, Behaviours and Epigenetic mechanisms, to Longevity Outcomes.

This was the most universal theme and was identified by sibling pairs as important in their survivor-hood in each of the 4 European countries, – with almost half of them commenting on this theme, often in concordant-pairs (Figure 1). Around this theme, nonagenarians reported “*being happy*,” “*always cheerful*,” “*never melancholy*” and having contentment with a “*rich life*” and family relationships “*thank God I have such good children*.” In an analysis of factors associated with health status, an “excellent/good” self-reported health also emerged as a predictor of survival in Italian GeHA nonagenarian siblings (Cevenini et al., 2013, 2014).

Typical examples from Northern Ireland include - Samuel at 95, who says that he remains

‘*interested in people, interested in life’*

**[Samuel, 95, NI]**

while sister Ethel at 93, says that being

‘*interested in things, and everything that’s going on’*

**[Ethel, 93, NI]**

Some Italian nonagenarians reported on their sense of positivity and good family relationships, in response to the question about survivorship. The positive attitude is clearly identified by Augusta who delights in saying how she feels at 95, and Virginia answers along the same theme

‘*I don’t feel melancholy. I don’t know what melancholy is*!’

**[Fausta, 95, Italy]**.

‘*I have always been happy*’

**[Virginia 97, Italy]**

In the Poland group, Krystyna at 92, one of two sisters, tells us in answer to the question of survivorship that her long life was related to

‘*My attitude to life, my cheerfulness. I am happy with everything*’

**[Krystyna 92, Poland]**.

Donata, 92, her sibling adds

‘*I am happy that I am with my daughter*’

**[Donata, 92, Poland]**

In Finland, there were very clear statements about having positive attitudes, with Matti at 91 telling us that his long life has depended on him being

‘*a joyful character, that does not worry unnecessarily*’

**[Matti 91, Finland]**.

#### Resilience

Managing and coping with difficulties seemed to be another common attitude which the 90-year-olds commented on either directly in response to the question about survivor-hood, or in their life story text. Many seemed to have an easy adaptability, willing to face new problems, acceptance of them and a determination to move on.

There were many examples which include Eileen.

Eileen at 96 demonstrates her adaptability in saying

‘[I] *just take things as they come*’

**[Eileen 96, NI]**.

And Krystyna, 92, from Poland, remembering and reflecting on her life and her traumatic war time experiences says with great emotion and feelings as she speaks about

‘*Being rounded up and taken to Pawiak jail, the Gestapo – all these things have passed and now everything is good*’

**[Krystyna, 92, Poland]**.

Maija 91, one of a trio of three sisters from Finland muses and responds

‘*You have to settle for what comes along and you will survive anything’*

**[Maija, 91, Finland]**.

and Hilkka, 95, her sister advises

‘*You must take things as they are and have a positive outlook’*

**[Hikka, 95, Finland]**

In Italy Giovanna 92 and her brother Dante, 96, in Italy reflect the same resilient type of personality

‘*You must think about the good side, believe, be strong and get over everything’* **[Giovanna 92, Italy]**

‘*Anything they give me, I will take*’

**[Dante, 96, Italy]**

And Assunta, 99, also in Italy and almost 100 year of age advises

‘*We solved problems in different ways’*……‘*we had to work and help bring money back because Father was ill*’

‘*I inherited this thing about working, keeping it together, being in a good mood*’

**[Assunta, 99, Italy]**

Her sister Antionetta 96, in Italy reflects

‘*Yes*…. *(we coped differently) because we needed to go to work*……*I don’t know what has helped me because I have gone through a lot of things*’

**[Antionetta, 96, Italy]**

Here around this theme, there seemed to be evidence of a nonagenarian’s locus of control, which seems to drive them forward with a sense of positivity irrespective of the obstacles and this is in keeping with the findings of Bowling and Iliffe (2011), “*about maximizing one’s psychological resources, namely self-efficacy and resilience,”* in their discussion about successful aging.

#### Social Networks

In the decade of Healthy Aging 2020–2030 (World Health Organization [WHO], 2020), the World Health Organisation has defined healthy aging; “it is the process of developing and maintaining the functional ability that enables wellbeing in older age” (World Health Organization [WHO], 2021). Social networks and interpersonal relationships have a crucial influence on health and there is much evidence connecting peoples’ social network and their health (Smith and Christakis, 2008; Goldsmith and Albrecht, 2011; Thoits, 2011). Strong social networks positively correlate with health whereas loneliness and reduced social contact undermined health behaviors with higher risk of mortality compared to people who have stronger and more satisfying support networks (Bowling and Grundy, 1998; Cohen et al., 2000; Kauppi et al., 2018).

Late life is a time often marked by a decline in personal health and increased need for social support. There may have been changes to peoples’ social lives, through loss of family members and friends, reduced functional abilities and constriction of their circle of autonomy, all of which can impact on health and wellbeing. Consequently, the social networks in which older people are embedded assume an increasingly central role in their health and wellbeing (Roth, 2020).

Reflecting on their families and their social contacts

Krystyna 92, from Poland, remembering her life and her traumatic war time experiences says with great emotion and feeling,

‘*I thank God that I have such good children and that my life was successful.’*

**[Krystyna, 92, Poland]**

Typical examples from Northern Ireland include - Samuel at 95, who says that he remains

‘*interested in people, interested in life*’

**[Samuel, 95, NI]**

And Sarah, 94, although not Samuel’s sibling, echoes exactly his reflections about maintaining interests

‘*keeping in touch with news and everything’*

**[Sarah, 94, NI]**

In Finland Hilkka talking about her what makes her happy says

‘*I am glad to see young and spirited people*

*-that gives me a lot of joy’ ‘And of course when the children come’*

**[Hikka, 95, Finland]**

**FIGURE 2:**
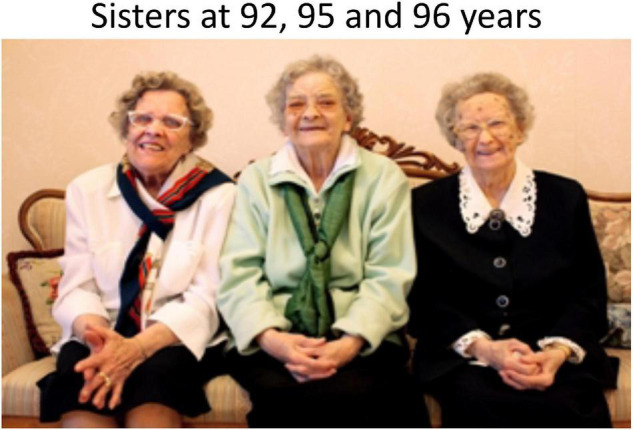
Hilkka, 95, Maija 93, Pulmu, 91 years of age. Three Finnish Nonagenarian Sibling Sisters-photograph by SAJR.

Maija, 91 and Hilkka, 95 and Pulmu, 93, three Finnish siblings, wished to be interviewed together and their conversation was a catch-up about their social activities as they lived a distance from each other (Figure 2) SAJR

‘*We are all widows, but we’ve been active. I have my friends and we have such a good time together when we go down to the sewing group of the Seafaring Association’*

**[Maija, 91, Finland]**

‘*How do you get ther*e’ Maija’?

**[Hikka, 95, Finland]**

‘*By bus’ ‘It’s always fun there and I really like going’ ‘All these things make me joyfu*l’

**[Maija, 91, Finland]**

Giovanna, 92, in Italy tells us

‘Happiness at home, when you’ve got all you need. I live alone but I always see my children’

**[Giovanna, 92, Italy]**

Adelma, 90, another sibling from Italy reports

‘*I’m happy I lived through all I did. First, I lived with my family and we were nine. Later the family grew to be 32 in all, what with grandparents and cousins living with us’*

**[Adelma, 90, Italy]**

Krystyna, 92, in Poland, explains about her social arrangements

‘*I don’t cook now because I have lovely neighbors who do it for me. I pay 15 zlotys for lunch and I’m delighted with it. I’m very happy.’*

**[Krystyna, 92, Poland]**

### Nonagenarian Sibling Relationships

‘*Sibling Relationships outlast marriages, survive the death of parents, resurface after quarrels that would sink a friendship. They flourish in a thousand incarnations of closeness and distance, warmth, loyalty and distrust*’ (Goode, 1994).

Siblings are a fixture in the family lives of children and adolescents (McHale et al., 2012), and there has been increasing research assessing how sibling hierarchy and relationships can affect behaviors in adulthood. In investigating sibling relationships in older adults and how those relations change across the life course, Gold (1989) classified the different types of sibling relationships as- Intimate, Apathetic, Congenial, Hostile, and Loyal. In the assessment of the ACUME2 sibling relationships, these classifications of the sibling relationships were explored to see if the Gold model was also applicable in nonagenarian siblings, and if differences were identifiable across the 4 European countries in which nonagenarian siblings lived and were interviewed.

#### Intimate Sibling Relationships

In this classification siblings tended to demonstrate a close relationship which transcended a sense of family obligation and was akin to a “*best friend*” relationship. The pair were most usually female and demonstrated a strong sense of emotional dependence There was often consistent and frequent contact between the siblings.

Sarah, Kathleen and Kelvin were a trio of living siblings in Northern Ireland (Kathleen’s twin Anne had recently died), who although the three did not live together or close by, managed to join together at the weekend, once a month to have a meal together. Speaking about their close family relationship during their lives, their reflections demonstrate an intimate-type-relationship that crossed over between the male and the female nonagenarians.

‘*We were ‘joined at the hip’ as they say – ‘we were always together’.’* ‘*I thought the world had ended when she died.*’

**[Kathleen, 94, NI, talking about sibling, Anne, who had recently died]**

‘*I am like my twin*…*We would just think the same or do the same sort of things. My twin was very close, very near*… *When Anne hurt her knee, I would be crying too*.’

**[Kathleen, 94, NI]**

‘*We are kind to each other*’

**[Kelvin, 92, NI]**

Siblings Krystyna, and Donata 92, who lived in Poland, had survived difficult times in Warsaw during the 2nd World War. They told a story of leaving Warsaw in a tank hidden beneath a blanket. Now in their nineties, and living some distance apart, they admitted that they were in contact daily.

Sibling Donata, 92, reports

‘*We talk on the phone five times daily. She knows everything about me*’

**[Donata, 92, Poland]**

And Krystyna, echoes the same sense of needing each other emotionally

‘*There is a strong bond between us. We call one another a few times a day, we get on well, never quarrel or get cross with one another*’

**[Krystyna, 92, Poland]**

#### Congenial Sibling Relationships

These relationships were characterized by mutual respect and a sense of a friendship which was more distant. A closer relationship of caring was reserved for, and only demonstrated at times of emotional stress and illness. However, these sibling pairs, maintained contact over months and years but did not necessarily see each other at high frequency.

Sarah and her sibling from Northern Ireland report

‘*We are all friendly. We see each other and talk*… *I go and see the rest of them, once a month, and we have a meal together. We are all quite easy going-never had any disagreements. Everyone minds their own business and that’s the best way.*’

**[Sarah, 94, NI]**

Maija, one of the Finnish sibling trio, remembering her family of seven children at home in younger days

‘*My sisters and brothers were always supportive, and the relations have been very good’*

**[Maija, 91, Finland]**

Norman, 95, and Joyce, 96, still living together in Northern Ireland reflect on their different ways of managing their congenial relationship

‘*Except for those few years when our work separated us, we were nearly together for all our lives. We had different opinions about things, but we learnt, just say nothing and go away from the problem’*

**[Norman, 95, NI]**

‘*Well, I don’t think there is a great resemblance in our outlook or actions, because we don’t discuss politics or life and things in general’*

**[Joyce, 96, NI]**

#### Loyal Sibling Relationships

These relationships showed a clearly defined responsibility based on family allegiance and obligation. The closeness was family-related and often idealized rather than shared at a deep emotional level. The relationships worked on a “*blood is thicker than water”* analogy, where nearness and physical proximity were more important than the actual psychological support provided.

Germana, in Italy reflects this type of loyal relationship with her sister

‘*We have different attitudes, my sister and me*… *We don’t have arguments, but she is different from me*… *When we were small, she felt responsible for me.*’

**[Germana, 96, Italy]**

Giovanna, describes a similar relationship about her very much older brother, who had already left home, when she was a very young child

‘*We are different*…*We’ve been apart so long, he’s got his own family, I’ve got mine.’*

**[Giovanna, 92, Italy]**

Matti in Finland reflects on his large family, where older children often left home to work on a neighbor’s farm or to live with relatives

‘*We never spent much time together. There were 12 children and so we were a good unit. We got on reasonably well.’*

**[Matti, 90, Finland]**

#### Apathetic Sibling Relationships

In these relationships there was no sense of a feeling of togetherness in the sibling life journey, or of sharing the journey emotionally. Although some siblings did not express indifferent feelings about their relationship, several siblings (one of the pair) as demonstrated below, tell of a detachment and lack of concern in sharing problems or seeing each other

Ethel with respect to her sister Maud

‘*Maud does not come to visit me. My family might take me there sometimes. Maud never cares if she never sees me. She is not interested in me*’

**[Ethel, 94, NI]**

Joyce and Norman, two male nonagenarian siblings, seems to be self-sufficient and not looking to have any help from each other

‘*Oh, we are different! But we are not stubborn.*’

**[Joyce, 96, NI]**

Joyce, seems to be self-sufficient and not looking to have any help from his brother

‘*Well, I don’t think there is a great resemblance in our action or our outlook’*

**[Norman, 95, NI]**

And Ellie, 100, too, reflects the same unwillingness to share problems and her reservation about discussing her worries

‘*We would both be reserved and would worry inside and carry it about. I don’t think we share problems very much, never did. I don’t discuss problems.*’

**[Ellie, 100, NI]**

Siblings from Poland both agree that they are not alike and do not share much of each other’s company

‘*We are not similar. Each of us is different’*

**[Janina, 93, Poland]**

‘*We have very little in common. My sister is less compassionate than me -more courageous’*

**[Stanislawa, 91, Poland]**

#### Hostile Sibling Relationships

Hostile-type relationships were also identified across the ACUME2 sibling pairs. These relationships were characterized by lack or closeness and sometimes actual enmity between the siblings, that had likely been triggered by past events. Envy or resentment stemming from childhood and family dynamics often played a large role in continuing confrontational episodes. Contact sometimes was avoided altogether.

Ethel and Maud two sisters in NI comment on their relationship as sisters

‘*I don’t know if they don’t like me; if they don’t they can lump it, that’s all I can say.*’

(**means** –‘I don’t know if they don’t like me; if they don’t that is their problem, that’s all I can say’)

**[Ethel, 94, NI]**

Maud is maybe not so much hostile as resentful about her life role viz a viz her sister Ethel; the relationship sounded rather frosty

‘*I would keep a problem to myself, whereas Ethel would have to be ‘top dog’ as I say, she would have to tell people everything*… *I was always the poor one in the family and still am! She’d always like to be dressed up’*

(**means** ‘I would keep a problem to myself, whereas Ethel would have to be superior one, as I would say; she would have to tell people everything … I was always the poor one in the family and still am! She’d always like to be dressed up’)

**[Maud, 94, NI]**

### What Coping Skills Did You Share?

Siblings play a unique role in one another’s lives and usually have shared more common life and cultural experiences across childhood and adolescent years, than any other relationship that has developed during later life, including between husbands and children. Examining sibling relations across the lifespan can increase the understanding of the social and cultural contexts in which lifelong sibling relationships are encouraged or hindered and may track with improved understanding of the psychological and life-style factors underpinning good quality aging, and longevity. An additional question “What coping skills do you share”? was posed to siblings either individually or together to further explore psychological factors which may have been important in the nonagenarian-sibling-relationship including coping in difficult times.

#### Italy

Giovanna, 92, and brother Dante, 96 give their views of how they coped in difficult times

‘*We had a pretty poor time during the Second World War and after it. But we had lots of affection between the whole lot of us -brothers and sisters- and we got on well. So, there was harmony and that helped. I am also a believer and I pray for everyone’*

**[Giovanna, 92, Italy]**

Dante, 96 years approached difficulties in different ways

‘*I worked. I’ve always worked, and I told the youngsters to work and get things done. Almost all of my family went to Mass, I still go to mass- I believe in God.*’

**[Dante, 96, Italy]**

Antionetta, almost 100 years of age, describes her coping mechanisms

‘*We solved problems in different ways. We were fond of each other and there wasn’t*

*much quarreling*’

**[Antionetta, 99, Italy]**

Assunta, 96, her sister, in response to the question ‘Do you and your sister share coping mechanisms’ says

‘*Yes*…

…*because I don’t believe much in anything, not even God….*’

**[Assunta, 96, Italy]**

#### Poland

Krystyna, 92, speaking about how she managed difficulties

‘*I somehow did not pay too much attention to my problems. I knew everything would be alright in the end. You could say it was Providence or something*’

**[Krystyna, 92, Poland]**

And reflecting on her sister Donata, and the support between them, Krystyna advises

‘*We stay in touch’*

**[Krystyna, 92, Poland]**

Sister Donata, who has had a stroke, and lives alone after her husband died, describes how she copes

‘*I do not worry too much’*

**[Donata, 92, Poland]**

And speaking about how she shares with her sister, Krystyna, advises

‘*She knows everything about me. We talk on the phone five times daily***’**

**[Donata, 92, Poland]**

Bronislawa, 93, and sister Marcjanna, 94, another Polish sibling pair, remember difficult times and how they coped during the war

‘*There were various situations that happened*… *but we coped with them somehow. I gave birth to my first child in 1940, just after the war started, and my second in 1943 around the time of the Warsaw Rising. We lived with other people as well, it was all difficult.*’

**[Bronislawa, 92, Poland]**

And speaking about her sister Marcjanna, Bronislawa remembers

‘*My sister has an even more difficult time than I did-her husband died in the Warsaw Rising and she had a miscarriage. Later she couldn’t carry babies- but then she was able to adopt a daughter’*

**[Bronislawa, 92, Poland]**

Marcjanna, herself told a little of her difficulties and how she managed

‘*The war was a tough experience and had a big influence on me-even bigger than the Warsaw Rising during which I lost my child and my husband.*’

‘*I somehow managed to go through them, I defeated everything that was bad; my faith helped me’*

**[Marcjanna, 94, Poland]**

Bronislawa speaking about when, and if the sisters will meet again, explains it is difficult, as they live far away,

‘*If one of us isn’t tired or ill, then the other one is-there is no opportunity*’

**[Bronislawa, 92, Poland]**

#### Northern Ireland

Mariah 92, and Sarah 93, who both lived separately, gave their views about how they have supported each other. Mariah, 95 first described something of their early life

‘*I have had so much trouble. My father went to sea- we hardly saw him. And then my mother took a stroke and we had to look after her. She was paralyzed and couldn’t do anything for herself, so we the family, had to look after her. But we were only children*,’

And replying about sharing and coping mechanisms with her sister, Mariah advises

‘*We never fall out over anything. I have to boss her!*’

**[Mariah, 92, NI]**

Sarah, 93, in replying, seems to suggest that Mariah has assumed a ‘mother-type’ role in her life

‘*I think Mariah is more like my mum-she does everything*’

‘*Oh yes, she looks after me*’

**[*Sarah*, 93, NI]**

#### Finland

Hikkla, 95, one of the trio of sibling sisters, talking about her relationship with her sisters and support between them says

‘*Just about, I think. We stick together quite well. We don’t quarrel much, and we have our own circle of friends*,…*all of our four brothers have died and two of our sisters too*’

**[Hikka, 95, Finland]**

Maija, 91, the second youngest also reflects on how the family loss and has affected them

‘*Now there are only four of us sisters alive*’

‘*We have always been supportive, and the relations have been very good*’

‘*Our youngest sister was a bit of a worrier, but the sisters are Christian and put their faith in God. I am a bit less religious, but I do say my prayers every evening. One simply must manage-there is no other option*’

**[Maija, 91, Finland]**

In our research, the most important resource which siblings recognized for improving their coping abilities was the affection and support within their siblinghood and this was present in all 4 European countries - Giovanna “*We had lots of affection between the whole lot of us*” in Italy; Krystyna in Poland, “*we stay in touch*”; Sarah in Northern Ireland, “*we never fall out over anything*”; and Hikkla, in Finland sums ups the general feeling of an important aspect of their intra-sibling coping by saying “*we stick together quite well*.” The siblinghood emotional ties, similar to the “intimate” Gold characteristic found between siblings, likely buffered their several types of severe emotional stress seen during the world war 11, of imprisonment, deprivation and forced removal from their country of birth. A further coping strategy finding was that siblings also looked outside their sibling and family relationships for support in difficult times and recognized their faith and prayer as an important aspect of their coping skills for instance Dante comments “*I believe in God*,” and Krystyna reports “*You could say it was Providence or something*,” whereas Maija in Finland reflects adding “*I say my prayers every evening.”* The feeling of strong resilient characters also emerged when the siblings were probed about coping abilities, with Maija adding “*One simply must manage-there is no other option*” Marcjanna telling us that “*I somehow managed to go through them*,” and Giovanna in Italy reporting “*Anything that they gave me, I will take*.” Other nonagenarians when faced with difficult times, immersed themselves completely in activity. Dante approached difficulties by reporting that “*I worked. I have always worked hard*” and likewise Adelma advises “*I like working, -I was born working and I will work until I die. I told the doctor to look inside the coffin in case I am still kicking! Bad weeds never die.”*

## Discussion

In our study of nonagenarian siblings in four European countries, the nonagenarian siblings self-identified optimism, adaptability, resilience, family nurturing and social networks, as important personal psychological characteristics that had contributed to their long lives.

**Optimism** was the most universal theme and was identified as an important psychological feature in their survivor-hood in each of the European countries, with almost half of the siblings commenting on this theme, often in concordant pairs. Optimism suggests that good things are likely to happen and that the future will be good; optimistic individuals feel more in charge of the goals and outcomes important to them, which fits with an increased sense of self-efficacy and control.

Scientific evidence is demonstrating that optimistic individuals are more likely to live longer and beyond 85 years of age. Two recent 10–30-year follow-up studies of more than 70,000 individuals mostly women, demonstrated that those with a more optimistic outlook, lived 11–15% longer lives, when risk factors such as smoking, demography and education were taken into account (Kim et al., 2017; Lee et al., 2019). Smaller studies have consistently shown that the most optimistic individuals seem to have improved resilience, which may give them a better ability to adapt to stressful or traumatic events (Ryff et al., 2004; Rasmussen et al., 2009; He et al., 2013; Sardella et al., 2021), and better response to chronic medical conditions (Gison et al., 2014). There is growing evidence to link a positive outlook on life with improved health from cardiovascular disease and diabetes (Giltay et al., 2004; Steptoe et al., 2010; Boehm et al., 2015; Kim et al., 2017). Research has also examined differences in immune response and wellbeing (Fancourt and Steptoe, 2020). There is evidence of stronger immune responses among optimists (Kohut et al., 2002), with individuals achieving seroprotective levels of influenza antibody having higher optimism, less anxiety, and lower perceived stress than the non-responders (Hayney et al., 2014).

Several studies suggest that resilient elderly people were more likely to experience positive emotion and optimism (Ong et al., 2006), which may promote health and longevity (Levy et al., 2002; Giltay et al., 2004, 2007; James et al., 2012), and reduce the risk of longer-term depressive symptoms (Giltay et al., 2006). Optimism could exert its beneficial health effects through personal engagement in health-promoting behaviors such as improved diet choices and abstinence from smoking (Giltay et al., 2007; Steptoe et al., 2010, 2015). Conversely higher levels of neuroticism or lower levels of conscientiousness or extraversion may be risk factors for the onset or progression of frailty (Gale et al., 2017). Some evidence also relates optimism to improved social relations, suggesting that optimistic individuals work harder at maintaining wider social networks (Srivastava et al., 2006; Smith and Christakis, 2008), with evidence that strong social networks can also enhance optimism (Segerstrom, 2007). These studies have a strong public health relevance because they suggest that optimism may be a modifiable psychosocial asset with the potential to improve health and extend human healthspan and lifespan (Smith and Hollinger-Smith, 2015; Malouff and Schutte, 2016).

Optimism has a likely genetic heritability of about 30%, with family studies demonstrating association between optimism levels in parents and children in two separate cohorts (Mosing et al., 2012; Rius-Ottenheim et al., 2012). This would be of particular interest in nonagenarian sibling pairs or trios, where our research found that >50% of sibling pairs, across the four countries, were congruent in self-reporting optimism as an association with their longevity. One study suggested that the oxytoxin receptor gene might have some relationship with optimism, but this finding was not replicated (Saphire-Bernstein et al., 2011; Cornelis et al., 2012). Early childhood experience has been linked to adult optimism and wellbeing (Heinonen et al., 2006; Kok et al., 2017) but it is not established whether optimism could have been cultivated directly through parental modeling or by instruction demonstrating coping success, thereby laying foundation for further optimism. It would be of interest to explore these possibilities in future research.

### Resilience and Adaptability

Managing and coping with difficulties seemed to be another common attitude which the nonagenarians commented on either directly or in response to the question about their survivorhood or reported in their life-story, in all the four European countries. Resilience has been defined as the process of adapting well in the face of adversity, trauma, tragedy, or major sources of stress, such as family and relationship problems, serious health problems, or workplace and financial stressors. A very large number of psychological factors have been identified as related to coping (Carver and Connor-Smith, 2010). The effectiveness of individual coping is said to depend on the type of stress, the individual and the nature of the social environment and is thought to be partially controlled by personality. Researchers are increasingly interested in measuring resilience and assessing its relationship to health behaviors (Lamond et al., 2009; Perna et al., 2012; Varadhan et al., 2018; Wu et al., 2020; Taylor and Carr, 2021; Zapater-Fajari et al., 2021). Previous research has shown that low psychological resilience is related to mental health issues such as anxiety and depression (Morete et al., 2018).

### Resilience and Coping

Resilience has been viewed as an important way of coping. Through resilience, an individual recovers and achieves emotional stability and avoids damaging outcomes from stressful situations (Leipold and Greve, 2009). Coping is defined as what people do to try to minimize stress. The stress and coping theory aligns with the buffering hypothesis; it states that social support protects people from the bad health effects of stressful events by influencing thought and coping ability. A number of studies have shown that coping style is associated with psychological health and well-being and moderated the negative effect of chronic illness among elderly people aged 60+ (Windle et al., 2008). The 2002–2005 Chinese study based on longitudinal data and using a resilience scale (Connor and Davidson, 2003), suggested that better resilience reduced mortality risk among the oldest-old in China (Shen and Zeng, 2010), and suggested that policies aimed to promote resilience could improve the quality of longevity in older people. In our nonagenarian sibling study women tended to report more stressful event occurrences, similar to previous research findings (Ong et al., 2006; Rubio et al., 2016). Although, the number of male nonagenarians was small compared to women, men mostly used problem-based or denial-type coping strategies compared to female nonagenarian siblings where emotional communication strategies with family and friends were used to help cope with stressful difficult life events (Meléndez et al., 2012; Herrera and Fernández, 2020).

Many of the nonagenarian siblings had seen profound changes in their lifetimes in the world in which they lived, the trauma of two world wars, the displacement of families in Poland, Finland, and Italy and during the Troubles in Northern Ireland, the death of husbands, wives, fathers, mothers and children, and the loss of homes and work. Despite these life events, nonagenarian life stories tell of a resilience, adaptability and a willingness to carry on. Maud 95, epitomizes the resilient nonagenarian personality and explains

‘*Oh, there was nothing to come in my way. I took everything in front of me, brushed it off and never let on it was there-keep going, keep going, keep going*’

(**Means -***and never let on it was there* …. and pretended it was not there’)

**[Maud, 95, N Ireland]**

demonstrating that their typical style of coping and their resilience did not seem to diminish with aging. Similarly, Kessing et al. (2003), in a Danish older population group and Tibubos et al. (2020), in a German study, found little evidence that accumulated stress events increased depressive symptoms throughout life. Searching and considering reasons why people should demonstrate higher wellbeing with increasing age, a hypothesis has emerged suggesting that as people age they accumulate emotional wisdom that leads to selection of more emotionally satisfying events, friendships and experiences, so that despite losses through the death of loved one, loss of status at retirement and loss of financial security, older people maintain or even increase wellbeing by concentrating on a more limited set of social contacts and experiences. Our findings are in keeping with the concept that how people face their aging is very important and that many successful agers use an adaptive optimizing coping strategy, rather than a focus on aging-related impairments or accumulated stressors (Baltes, 1993; Bandura, 1997; Kahana et al., 2014).

### Sibling Relationships and Coping Strategies

The most important resource which siblings recognized for improving their coping abilities was the affection and support within their siblinghood and this was present in all four European countries.

In Finland, Hikka, summed up the general feeling of an important aspect of their intra-sibling coping by saying

‘*we stick together quite well.*’

**[Hikka, 95, Finland]**

The siblinghood emotional ties, similar to the ‘intimate’ Gold characteristic found between siblings, likely buffered their several types of severe emotional stressors suffered during the world war 11, of imprisonment, deprivation and deportation from their country. A further coping finding was that siblings also looked outside their sibling and family relationships for support in difficult times and recognized their faith and prayer as an important aspect of their coping abilities, for instance, Dante and Krystyna comment

‘*I believe in God*,’

**[Dante, 96, Italy]**

‘*You could say it was Providence or something.*’

**[Krystyan, 92, Poland]**

Other nonagenarians when faced with difficult times, immersed themselves completely in some diversionary but self-fulfilling, coping activities, such as Matti or Adelma who advised

‘*I worked. I have always worked hard*’

**[Matti, 96, Italy]**

‘*I like working, -I was born working and I will work until I die. I told the doctor to look inside the coffin in case I am still kicking! Bad weeds never die.*’

**[Adelma, 96, Italy]**

Most nonagenarian sibling relationships when analyzed using the Gold classification fitted the “congenial” or “loyal” relationship type – demonstrating a healthy respect for the other’s opinion without overt dependence. Few fitted either the “Intimate,” “apathetic” or “hostile” type relationships. There were different cultural European influences. Siblings in Italy and Poland were more likely to report supportive siblinghood, compared to pairs/trios in Finland or Northern Ireland where self-resilience and independence seemed more common. Polish and Italian nonagenarians often felt more supported by their religious faith and church. Most male/female dyads demonstrated a healthy respect for each other’s opinion and their sibling relationship fitted the “*loyal*” type, though with a clear sense of independence. Several of female/female, but no male/male dyad fitted the “*intimate*” description and two might be described as “*apathetic.*” The most common loyal and congenial nonagenarian sibling relationships may help the sibling’s individual coping and survival mechanisms. However, perhaps these 90-year-olds survive because they demonstrated resilient and independent personalities and don’t need to depend on each other.

According to Goode (1994), “*Sibling relationships – outlast marriages, survive the death of parents, resurface after quarrels that would sink any friendship. They flourish in a thousand incarnations of closeness and distance, warmth, loyalty and distrus*t.” A sibling’s position in the family gives rise to social psychological processes, with lifelong implications for the development and adjustment of each brother or sister (Irish, 1964). A recent longitudinal study investigating the effect of empathy found that both older and younger siblings during childhood can positively influence each other’s empathic concern for others, over a lifetime (Jambon et al., 2018). Emotional stability and conscientiousness can influence health-related behaviors and age-related physiological health and the increased chance of reaching an advanced age (Terracciano et al., 2008; Milad and Bogg, 2020), whereas stressful life events, depression and personality traits such as low agreeableness, low conscientiousness, high openness and high neuroticism can have a negative effect on health (Mitchell et al., 2021). The nonagenarian sibling group generally demonstrated a good measure of consolidated support between the siblings, and there was evidence that this often began in childhood where families had experienced a loving and nurturing family life. Because of demography and social trends, women nonagenarians seem more likely to sustain more active sibling relationships with sisters, or single siblings, compared to male nonagenarians.

### Mechanisms Related to Resilience and Social Networks

The four-country nonagenarian sibling group generally demonstrated both intra-sibling but also family and community networks of support. Some large intergenerational nonagenarian families who had lived together during difficult wartime, deportment, or other family crises continued to provide support at times death, illness and loss of autonomy. Most nonagenarians were still involved in community networks, such as Hikka, and her sisters in Finland involved in the local Sea-Faring Association, whereas, Samuel and Sarah in Northern Ireland continued to write books about their life stories and were involving themselves in charity events. Social networks and interpersonal relationships have a crucial influence on health and there is much evidence connecting peoples’ social network and their health (Smith and Christakis, 2008; Goldsmith and Albrecht, 2011; Thoits, 2011; Dumitrache et al., 2017, 2019), whereas loneliness and reduced social contact undermined health behaviors, with higher risk of mortality (Bowling and Grundy, 1998; Cohen et al., 2000; Kauppi et al., 2018). Two prospective studies have demonstrated that longevity in the oldest old was associated with good social integration and an optimistic personality (Lee et al., 2019; Trudel-Fitzgerald et al., 2019).

Understanding the molecular basis of how behaviors are established, maintained and altered by environmental factors is an area of growing interest. It has been long known that the brain responds to psychological stress by activating the sympathetic system in a ‘*fight and flight*’ response with the release of cortisol that can modulate neuronal circuits and brain plasticity. Psychological stressors such as social isolation contribute to poor psychological health, susceptibility to disease and psychiatric illness (Cuijpers et al., 2014; Xiao et al., 2015; Pietrabissa and Simpson, 2020). Research finds higher stress hormone levels in lonely and socially isolated people (Dalagard et al., 2008; Doane and Adam, 2010; Cacioppo et al., 2015), with evidence of methylation change in the glucocorticoid receptor in relation to life adversity (Daskalakis and Yehuda, 2014).

According to the World Health Organization (World Health Organization [WHO], 2020), Healthy Ageing (HA) is the “process of developing and maintaining the functional abilities that enables well-being in older age” (World Health Organization [WHO], 2021). Successful aging is considered to be more than the “absence of disease and maintenance of high functioning” (Baltes and Baltes, 1990), but involves the active engagement in everyday social activities. The value and number of social interactions daily and inclusion in family networks seems to make for good mental health and social well-being (Rowe and Kahn, 1998). Around this theme, nonagenarians reported “*being happy*,” “*always cheerful*,” “*never melancholy*” and having a contentment with a “*rich life*” and family relationships “*thank God I have such good children*.” Social networks and supportive relationships can buffer the effects of stressful life events such as bereavement, deteriorating health and loss of autonomy and mitigate negative feelings and emotions. These findings of optimism and positive attitude to life, fit with accumulating evidence that the biomedical health model does not effectively assess the quality of life in older age, but rather life satisfaction comes from a perceived self-efficacy and optimism (Strawbridge et al., 1996). Bowling and Iliffe report similar findings from a UK postal study of people, aged 65–75, where respondents reported that for them “*successful aging was not only about the maintenance of health, but about maximizing one’s psychological resources, namely self-efficacy and resilience*” (Bowling and Iliffe, 2011). Stable loving family life and social relationships are increasingly recognized as highly important in child development and in protection from depressive and anxiety illness in adult life (Nanni et al., 2011). The nonagenarian life stories, told of shared childhoods in large families, often with a firm discipline, but with a central mother figure and a secure loving environment. Virginia at 97 identifies exactly this theme and the important role of her mother and family love in determining the quality and length of her life, when she succinctly responds

‘*Because our mother loved us. I have been happy*’

**[Virginia, 97, Italy]**.

### Limitations and Strengths

The authors accept that recontacting the GEHA nonagenarian siblings, may have amplified a good attitude and contributed to the outcomes of optimism, but great care was taken to reduce any compromise of the data collected, for instance several interviewers were medical students not previously known to the GEHA nonagenarian participants. The interviews and photographs took place at different times of the year, snow in Poland and summer heat in Italy, because of the difficult logistics in organizing appointments with both nonagenarians and the training and availability of interviewers and the photographer. All interviewers used the same protocol and were instructed to stay with the script. Bias was reduced in the data interpretation by having independent language translators and two researchers coding data in different geographical sites. The main strength of our research findings was that the psychological features of optimism, resilience and social networks were self-reported by nonagenarian siblings as important factors in their longevity, and that the results emerged independently from four countries with different cultural norms and beliefs, in the north, south, east and western poles of Europe. In addition, our findings aligned with other research studies in oldest old cohorts. The GeHA research cohort is also unique in that the participants are nonagenarian siblings and both sexes reported similar longevity beliefs. However, our research involves a small number of subjects and the analyses may not be generalizable and require replication in other sibling nonagenarian groups, to better substantiate the findings.

## Conclusion and Future Directions

Through their “voices” in text, we hear something of how GeHA nonagenarian siblings understood their survivor-hood and longevity. A majority of the nonagenarians showed a positive optimistic psychological attitude to life twinned with a self-deprecating sense of humor, and for many, these traits were accompanied by a feisty independence. There was also evidence of a resilient and adaptive approach to life, across all countries, although coping adaptive strategies tended to differ. There was an almost universal agreement that nonagenarians enjoyed the “love and support of their families and children” and that was their one wish for their children too. Keeping in touch and maintaining interests in family and social networks were universally important themes. Optimism, resilience, adaptability and a feisty independence seemed to come across as the main features of the psychological profile of the GeHA nonagenarian sibling, irrespective of their country of origin. The nonagenarian siblings are living examples of how a combination of factors (Figure 1), family genes, psychological behaviors and social networks, embedded in an optimistic, adaptive and resilient personality seem to have improved their chance of living longer and with a better quality of life.

The most common loyal and congenial nonagenarian sibling relationships across the four-countries, may help the sibling’s individual coping and survival mechanisms. Siblings in Italy and Poland were more likely to report supportive siblinghood, compared to pairs/trios in Finland or Northern Ireland where self-resilience and independence seemed more common. Polish and Italian nonagenarians often felt supported by their religious faith and church. Increasing knowledge and understanding about physical and mental resilience have strong public health relevance because there is evidence that optimism and resilience may be modifiable and teachable psychosocial assets, with the potential to improve health and extend human healthspan and lifespan.

## Data Availability Statement

The raw data supporting the conclusions of this article can be made available with agreement of the authors.

## Ethics Statement

The studies involving human participants were reviewed and approved by the Research Ethics Committee Northern Ireland (ORECNI), 08/NIR03/42 and Queens University of Belfast. The patients/participants provided their written informed consent to participate in this study. Written informed consent was obtained from the individual(s) for the publication of any potentially identifiable images or data included in this article.

## Author Contributions

IR and SR conceived and designed the research and the outline of the manuscript. IR, SR, KB, ES, EC, LC, JR, VF, and CF contributed to ACUME2 research design, collection of materials, the analysis of the data, and approved the various iterations of the manuscript prior to submission. All the authors contributed to the article and approved the submitted version.

## Conflict of Interest

The authors declare that the research was conducted in the absence of any commercial or financial relationships that could be construed as a potential conflict of interest.

## Publisher’s Note

All claims expressed in this article are solely those of the authors and do not necessarily represent those of their affiliated organizations, or those of the publisher, the editors and the reviewers. Any product that may be evaluated in this article, or claim that may be made by its manufacturer, is not guaranteed or endorsed by the publisher.
